# Adverse Drug Event Discovery Using Biomedical Literature: A Big Data Neural Network Adventure

**DOI:** 10.2196/medinform.9170

**Published:** 2017-12-08

**Authors:** Ahmad P Tafti, Jonathan Badger, Eric LaRose, Ehsan Shirzadi, Andrea Mahnke, John Mayer, Zhan Ye, David Page, Peggy Peissig

**Affiliations:** ^1^ Biomedical Informatics Research Center Marshfield Clinic Research Institute Marshfield, WI United States; ^2^ Institute of Electrical and Electronics Engineers Dublin Ireland; ^3^ Department of Biostatistics and Medical Informatics University of Wisconsin-Madison Madison, WI United States

**Keywords:** adverse drug event, adverse drug reaction, drug side effects, machine learning, text mining

## Abstract

**Background:**

The study of adverse drug events (ADEs) is a tenured topic in medical literature. In recent years, increasing numbers of scientific articles and health-related social media posts have been generated and shared daily, albeit with very limited use for ADE study and with little known about the content with respect to ADEs.

**Objective:**

The aim of this study was to develop a big data analytics strategy that mines the content of scientific articles and health-related Web-based social media to detect and identify ADEs.

**Methods:**

We analyzed the following two data sources: (1) biomedical articles and (2) health-related social media blog posts. We developed an intelligent and scalable text mining solution on big data infrastructures composed of Apache Spark, natural language processing, and machine learning. This was combined with an Elasticsearch No-SQL distributed database to explore and visualize ADEs.

**Results:**

The accuracy, precision, recall, and area under receiver operating characteristic of the system were 92.7%, 93.6%, 93.0%, and 0.905, respectively, and showed better results in comparison with traditional approaches in the literature. This work not only detected and classified ADE sentences from big data biomedical literature but also scientifically visualized ADE interactions.

**Conclusions:**

To the best of our knowledge, this work is the first to investigate a big data machine learning strategy for ADE discovery on massive datasets downloaded from PubMed Central and social media. This contribution illustrates possible capacities in big data biomedical text analysis using advanced computational methods with real-time update from new data published on a daily basis.

## Introduction

### Background

Adverse drug events (ADEs), defined as the set of detriments or injuries caused by a medication, have led to additional medical costs, prolonged hospitalization, morbidity, and ascribable disability worldwide [1-4]. ADEs encompass all adverse drug reactions but also include preventable causes of errors such as inappropriate dosing, dispensing errors, and drug abuse. Discovery of ADEs has gained great attention in the health care community, and in the last few years, several drug risk-benefit assessment strategies have been developed to analyze drug efficacy and safety using different medical data sources, ranging from electronic health records (EHRs) to human-health–related social media and drug reviews [5-14]. A variety of combined computational methods using natural language processing (NLP), machine learning strategies, and text retrieval algorithms have been employed to extract ADEs from such data sources [15-23]. Clinical trials, EHRs, and medical case reports are additional biomedical data-rich sources that have been utilized for ADE extraction [24-28].

**Figure 1:**
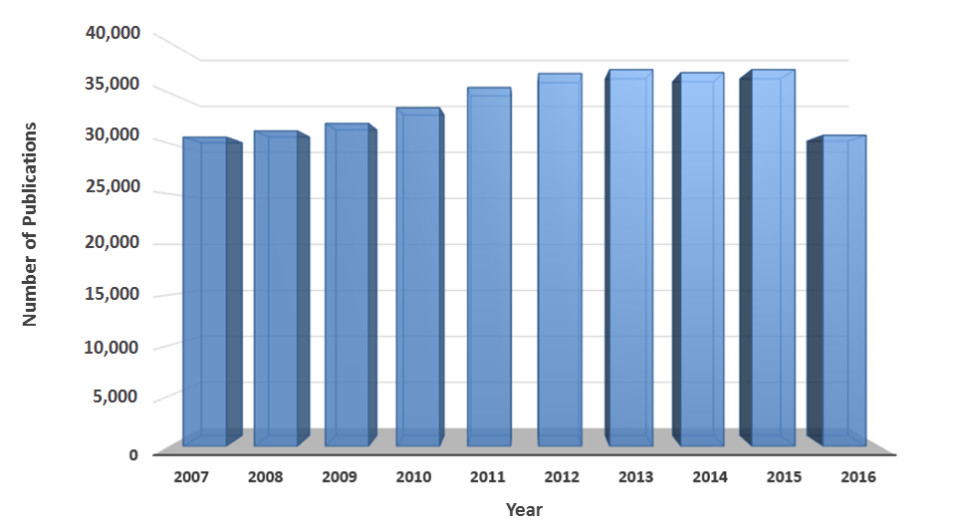
The number of publications in several medication studies available at PubMed over the last 10 years. The results obtained by submitting a query: ((((((((((drug analysis[MeSH Terms]) OR drug analysis[MeSH Subheading]) OR adverse drug event[MeSH Terms]) OR adverse drug event[MeSH Subheading]) OR adverse drug reaction[MeSH Terms]) OR adverse drug reaction[MeSH Subheading]) OR drug evaluation[MeSH Terms]) OR drug evaluation[MeSH Subheading])) OR drug repositioning[MeSH Terms]) OR drug repositioning[MeSH Subheading].

In recent years, biomedical articles produced by scientists all across the world have grown extensively. Figure 1 [29] shows that the number of journal and conference papers published in different medication studies (eg, ADEs and drug analysis, drug evaluation, and drug repositioning) rapidly grew in number from year 2007 to 2016. The total number of publications in those years is approximately 342,301 articles. To roughly estimate the size of such scientific papers, we assumed a PDF file format for each article. The size of a PDF file depends on the number of pages and pictures or metadata inside the file. Considering a 9 to 11 page PDF including plain text along with a few pictures, it may equal almost 3 MB size in average, and it appears, approximately 1.02 TB articles were generated in drug associated studies from 2007 to 2016. The other file formats such as extensible markup language (XML), may be much larger in size. Scientific articles published in biomedical research are usually generated using standardized and principled methods and therefore, are especially valuable for high-quality knowledge discovery. This great deluge of information includes an enormous number of scientific publications on ADEs’ study, an area of focus into which many biomedical researchers have entered, developing a variety of research activities for discovering, analyzing, and monitoring ADEs [30-38].

It is impossible for researchers, scientists, and physicians to read and process the large body of scientific articles and remain abreast of the foremost information regarding ADEs. Therefore, there is a pressing need to develop intelligent computational methods, particularly big data analytics solutions, to efficiently process this wealth of data. Big data biomedical text analysis utilizes advanced computational technologies including big data infrastructure, NLP, statistical analytics, and machine learning algorithms to extract facts from text data. This in turn generates new hypotheses by systematically analyzing large numbers of scientific publications.

### Objectives and the Main Contributions

Whereas ADE discovery from diverse biomedical data sources in general has been studied historically in health care informatics, the use of big data scientific articles and health-related social media for ADE discovery has been very limited so far. The motivation of this work is to study big data machine learning solutions, particularly big data neural networks (bigNN), to analyze ADEs from large-scale biomedical text data, developing a scalable framework to fulfill the following objectives: (1) to extract current knowledge and high-quality information about ADEs using full text scientific articles and social media, (2) to utilize and adapt advanced NLP and machine learning algorithms in a large-scale fashion by the use of big data infrastructures, and (3) to provide better insights and tendencies in large-scale biomedical text analytics and identify the challenges and potential enhancements toward efficient and accurate ADE discovery. We briefly summarize our *main contributions* as follows:

We initiated a study of big data literature mining for ADE discovery with the use of two different data sources: (1) published full text scientific articles available on PubMed Central [39], and (2) posts available in health-related social media, including MedHelp [40], patient [41], and WebMD [42]. Although several promising approaches have been designed for biomedical text mining, the development of scalable machine learning frameworks capable of ADE extraction from big data is very limited. To the best of our knowledge, our work is the first to investigate a bigNN strategy for ADE discovery on massive datasets downloaded from PubMed Central and social media.

- With the current work and using big data analytics platforms such as Elasticsearch and Apache Spark, we developed a scalable framework to analyze and visualize ADEs from hundreds of thousands of published scientific articles and social media blog posts.
- Combining a variety of the internal neural network parameters, we presented a predictive model that obtained accuracy, precision, recall, and area under 92.7%, 93.6%, 93.0%, and 0.905, respectively, on a massive dataset downloaded from PubMed Central plus health-related social media.
- This paper opens the door to pursue large-scale biomedical literature mining and its application in health care informatics in general and introduces several possible enhancements to advance the level of the impact of this research area.

## Methods

**Figure 2:**
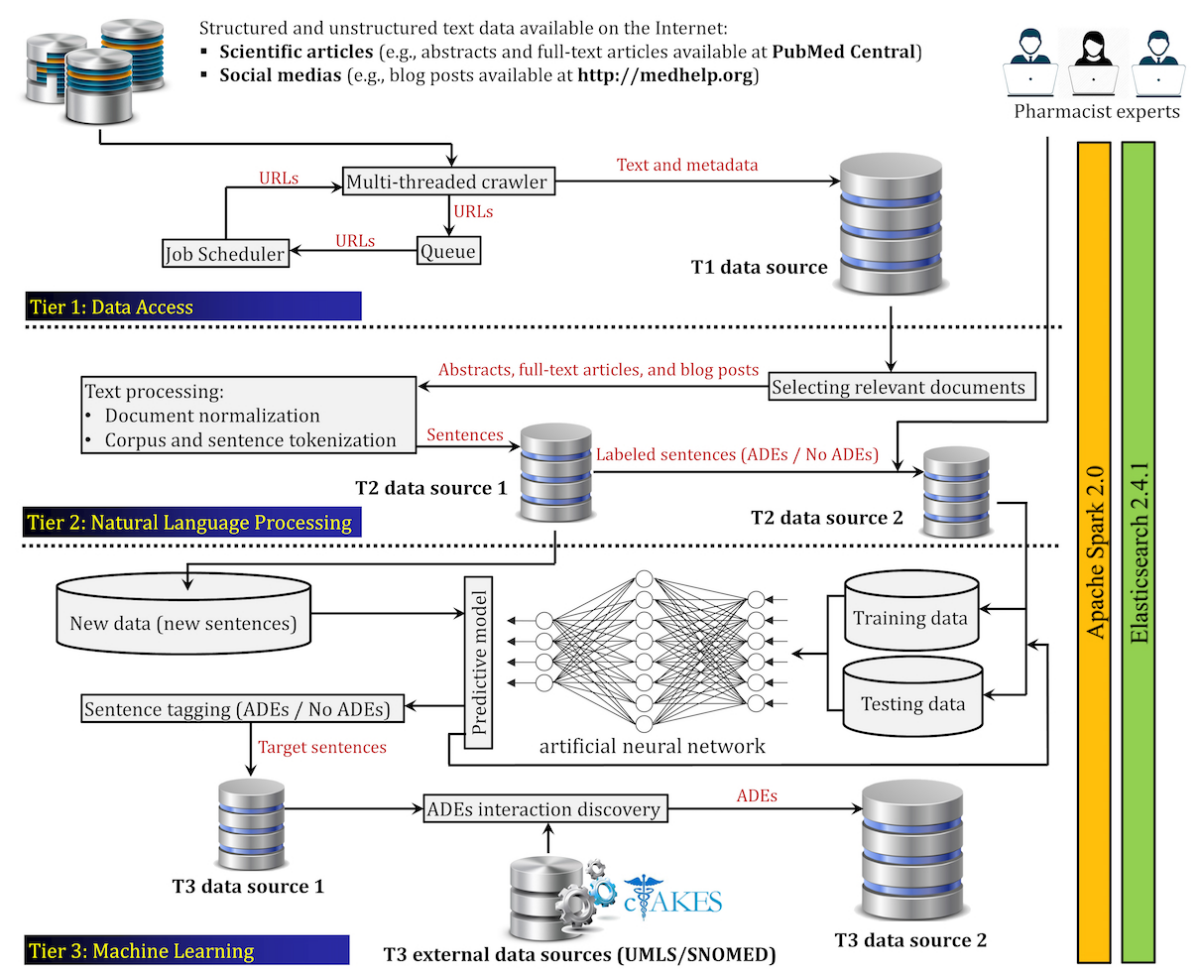
The proposed system for adverse drug event (ADE) discovery. All tiers developed on top of the Apache Spark 2.0 that utilizes an Elasticsearch database 2.4.1 to data storage and retrieval.

The general pipeline of the proposed ADE extraction framework is illustrated in Figure 2. In this section, we shall explain the underlying tiers of the proposed bigNN framework.

### Tier 1: Data Access

Tier 1 systematically collects the expanding body of scientific articles and social media blog posts through different data sources available on the Internet. A multi-threaded crawler or downloader was developed to provide timely and efficient processing of the diverse big data content found on the Internet. *Scrapy* [43], a free and open source Web crawling system has been used to allow multiple threads to automatically fetch URLs from different sources. A queuing system and a scheduler have also been established as a part of the data access tier.

All collected data (eg, XML files) are first turned into plain text data, and together with associated metadata (eg, journal name, author list, and publication date) are stored in a single type (table type) inside a No-SQL database, namely Elasticsearch [44], which provides a distributed, open source, RESTful and full text search and analytics engine.

### Tier 2: Natural Language Processing

Tier 2 includes several computational procedures to process raw text data, preparing potential ADE sentences to feed the next tier.

#### Selecting Relevant Documents

One of the major goals of the proposed system was to collect groups of sentences that provide evidence about drug-event pairs. Toward that outcome, there is an emergent need to identify trustworthy and reputable data sources (eg, well-founded and prestigious journals such as *Nature*, *PNAS*, and *PLOS*). As the proposed framework accumulates data from two separate data sources, including scientific journals and messages posted on social media, we established two different criteria to yield more credible data. Section A.1 of Multimedia Appendix 1 further discusses the proposed method and criteria.

#### Text Processing

We first normalize all documents by converting corpora into a standard consistent form. This process (1) converts all characters to lower case, (2) transliterates to American Standard Code for Information Interchange if needed, and (3) deletes a set of existing substrings and patterns (eg, [], , ?, !, and ()). Once we complete the proposed text normalization process, we convert every document into a set of sentences. Although several ADEs could be captured among different sentences, extracting ADEs interactions across sentences is significantly more challenging than within sentences [6,45]. To feed the bigNN system, a random subset of sentences was selected for manual annotation by three domain experts; see Section A.2 of Multimedia Appendix 1 for details. The random subset of sentences includes health and medical-related text data either with or without ADE interactions. Sentences that are missing either a drug name or an adverse drug effect term were excluded. Using a Web application (Multimedia Appendix 1), the domain experts labeled individual sentences as ADEs, No-ADEs, or Not Decided. To focus on binary classification, we omitted sentences labeled “Not Decided” leaving two different classes: (1) ADEs and (2) No-ADEs. Section A.2 of Multimedia Appendix 1 explains how we made a training set for the machine learning tier.

### Tier 3: Machine Learning

Tier 3 implements the core functionality needed for making a predictive model to distinguish ADEs sentences versus those that are No-ADEs. From the machine learning perspective, this means making a binary classification predictive model that assigns one of two trained labels to new unlabeled sentences. Therefore, when an arbitrary sentence arrives, the predictive model chooses one category exclusively from two predefined classes as ADEs or No-ADEs. There are two basic steps. The first step is to extract effective content as a set of features. The next step is the text classification assignment. The bag-of-words (BoW) representation, a widely used content extractor algorithm, has been around for several years in the text analytics domain, and it provides an easy way to turn text-based data records into a set of feature vectors such that the frequency of occurrence of words (eg, uni-grams and/or bi-grams, along with part-of-speech [POS] tagging) in the corpus is used as a feature vector to train a classifier (eg, support vector machine [SVM], decision tree, and/or logistic regression) [46-51]. The BoW representation maintains word intensities across the corpus, but it dissembles grammar, syntactic, semantic, and word order. In contrast to the BoW representation, the word2vector (word2vec) algorithm, originally developed at Google [52], includes a set of computational methods that turns a corpus of text data into a meaningful vector space that encompasses grammar, semantic, and word order. Word2vec comes with a two-layer neural network and is able to tackle several text analytics functions including dependency parsing [53,54], named entity recognition [55,56], text classification [57,58], and word clustering [59]. The model takes a text corpus as an input and turns each word in the corpus into a vector as illustrated in Section A.3 of Multimedia Appendix 1. It then groups vectors of similar words together in a vector space, training words against other words that neighbor them in the input text corpus [60,61]. Our bigNN system implemented the word2vec neural network, which is fully explained in Section A.3 of Multimedia Appendix 1.

**Figure 3:**
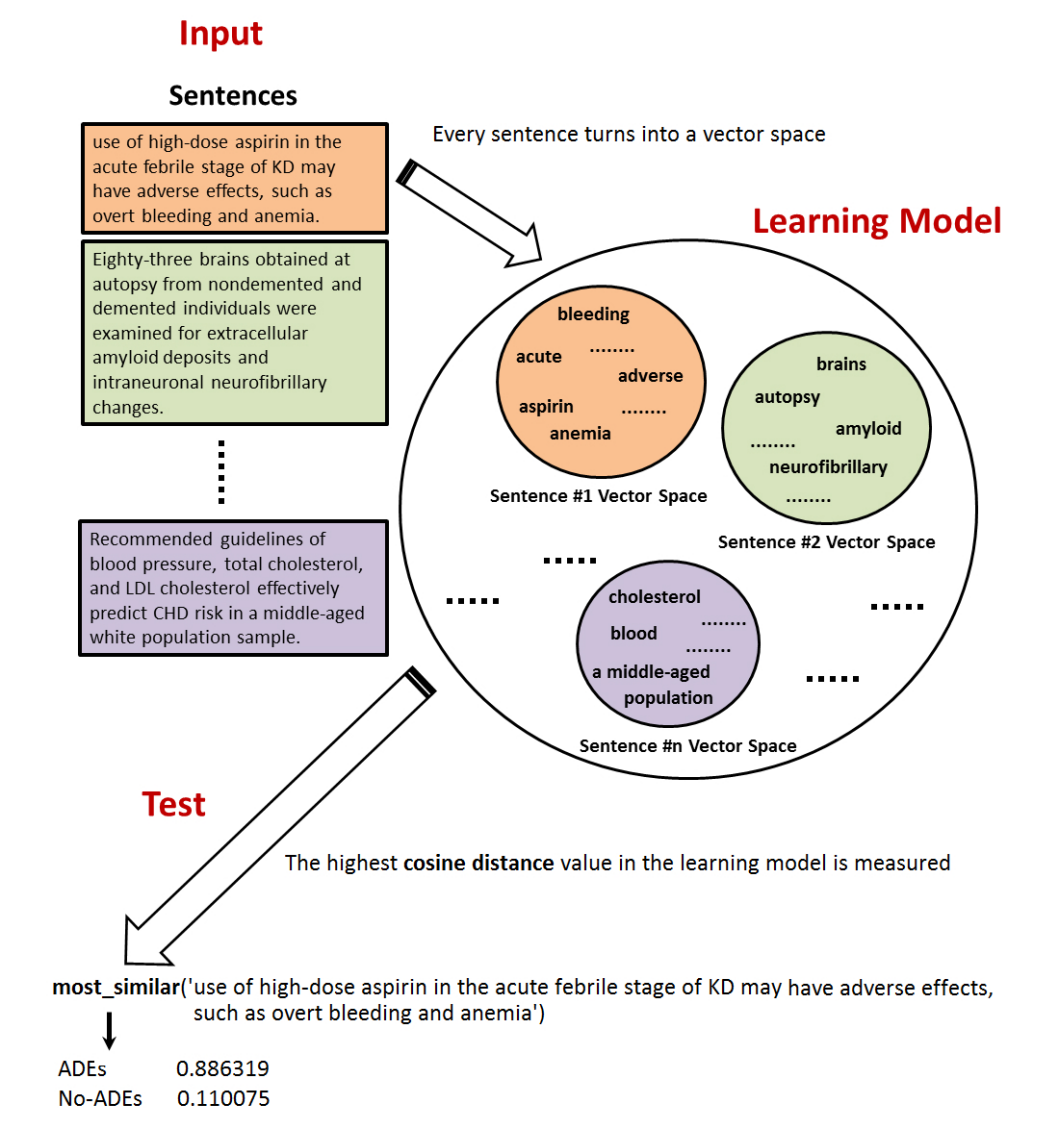
This figure depicts the abstract view of the proposed learning model where the cosine distance similarity is measured to select an appropriate category for a given sentence. Every single word is represented as a vector, and eventually every sentence (eg, adverse drug events [ADEs] or No-ADEs) turns in to a vector space. Once we have done with training the model, for every new coming sentence, the highest cosine distance value is then measured to find the best fitness class.

An abstract view of the proposed learning algorithm is shown in Figure 3. As mentioned in Section A.3 of Multimedia Appendix 1, the algorithm builds a vector representation for words and/or sentences. If we train a learning model with 98-dimension, then we will obtain a 98-digit in front of each word (eg, “aspirin”). The cosine distance similarity [62,63], which is the normalized dot product between vectors, is then measured to find the best fitness class for a sentence. Once we have the labeled sentences as ADEs or No-ADEs, we focus on ADE sentences and find the positive adverse-drug interactions using cTAKES [64].

## Results

### Implementation and Test Bed

We investigated two different data sources: (1) biomedical articles and (2) health-related social media blog posts. The first data source included 97,246,719 sentences obtained from almost 1,451,413 abstracts and full text articles available on PubMed Central, and the second one consisted of 2,524,622 sentences obtained from 419,915 blog posts at MedHelp, Patient, and WebMD. To train our proposed predictive model illustrated in Figure 2, we first randomly selected different subsets for the purpose of manual annotation. Those subsets were annotated by three domain experts who have been working in medical and pharmacy domains. We defined two different classes as ADEs and No-ADEs to indicate drug-event interactions in a sentence. The sentences that included positive drug-event interaction were tagged as ADEs, and the others were tagged as No-ADEs. Before the large-scale manual extraction, we evaluated the interrater reliability among our three domain experts, the results of kappa statistics, .84, which indicates very good concordance and agreement; more details are reported in Section A.2 of Multimedia Appendix 1.

**Table 1:** Nine datasets were employed to make and evaluate the big data neutral network (bigNN) system. Each row identifies the dataset along with the number of ADEs and No-ADEs sentences within the dataset. Every row also shows how many of these sentences are human-labeled and/or machine-labeled. The datasets are separated into three main categories: biomedical articles (*_BA), social media posts (*_SM), and the combination of the two (*_Combined).

Dataset ID	Total number of sentences	Number of ADEs^a^ sentences	Number of No-ADEs sentences	Number of human-labeled sentences	Number of machine-labeled sentences
ADEs#1_BA	6960	3311	3649	6960	0
ADEs#1_SM	400	160	240	400	0
ADEs#1_Combined	7360	3471	3889	7360	0
ADEs#2_BA	13,545	6359	7186	7015	6530
ADEs#2_SM	472	195	277	405	67
ADEs#2_Combined	14,017	6554	7463	7420	6597
ADEs#3_BA	21,278	10,307	10,971	7015	14,263
ADEs#3_SM	565	241	324	405	160
ADEs#3_Combined	21,843	10,548	11,295	7420	14,423

^a^ADEs: adverse drug events.

Next, we developed nine different datasets from those sentences to train the proposed bigNN system. The first set of datasets (the first three rows in Table 1) used only human-labeled datasets from our domain experts. Within these three datasets, three types of sentences were generated: the first type was biomedical articles, the second was social media, and the third was the combination of the two. For the second set of datasets (the three datasets in the middle of Table 1), we added some machine-labeled datasets (the number of datasets were reported in the last column, “number of machine-labeled sentences” of Table 1) with human-labeled datasets to increase the sample sizes. We also generated three types of sentences as illustrated above. For the third set of datasets (the last three rows of Table 1), we added in more machine-labeled datasets with human-labeled datasets; similar three types of sentences were generated.

We also utilized three smaller datasets, including 2600, 3500, and 4000 human-labeled data records and plotted learning curves to see how the accuracy of the predictive model varies with increasing amount of training data. The results were not promising enough, and we started with a larger human-labeled dataset as illustrated in the first three rows in Table 1. For all the experiment, we utilized 75% of every dataset to train the model and 25% to test it using four-fold cross validation, with no sentence to appear in both the training and testing sets at the same time.

### Experimental Setup

Every programming module in Tier 1 and Tier 2 was developed by Python 2.7.13. Tier 3, the machine learning tier, was implemented by Java j2SE 8. All of these tiers were developed on top of the Apache Spark 2.0 [65] and Elasticsearch DB 2.4.1 [44] just to tackle the problem of big data analytics in an efficient and timely fashion. From the computational side, a dedicated computational resource, including two virtual machines in a VMWARE cluster environment, each running a 64-bit CentOS 6.8 operating system with 8 vCPUs, 16 GB RAM, and 1 TB HDD in total, hosted on a Xeon E5-2690V3 2.6 GHz CPU, were used to obtain the experimental results.

### Experimental Validations

We analyzed the performance of the predictive model across all the datasets. Accuracy, precision, and recall obtained by the experiments are shown in Multimedia Appendix 2. The first column shows the dataset used to in the experiments. The second column describes a configuration setup for a set of internal parameters of the proposed neural network model. Minimum word frequency (MWF) allows for ignoring all words in the vocabulary with total occurrences lower than MWF value. Epoch (EP) is the number of forward and backward passes of all training examples. Window size (WS) defines context windows size to generate a vector representation for words across the documents. Iteration (ITR) defines the number of iterations done for each mini-batch during a training process. The last column shows elapsed time for the training stage. This does not reflect the time of text-preprocessing tasks such as normalization and tokenization. The current table shows that greater EP and ITR with the use of WS of two will provide better performance across all three datasets. To further analyze our proposed ADEs sentence discovery system, we also compared the proposed predictive model with the combination of BoW feature selection method and SVM, decision tree, and naïve Bayes classifiers. Uni-grams, bi-grams, along with POS tagging were used as BoW features to make a predictive model. One can see in Multimedia Appendix 2, the most promising accuracy results across all datasets obtained by the (MWF=2%, EP=25, WS=2, and ITR=10) configuration. Furthermore, we demonstrated that the performance of bigNN system, both accuracy and time of completing the task, is comparable with traditional SVM, naïve Bayesian, and decision tree with BoW strategy. The results of this experiment are shown in Table 2. All the measures in Table 2 are selected from the best performed model by tuning the models using different parameters for all the bigNN system and SVM, naïve Bayesian, and decision tree with BoW strategy. Regarding the BoW feature set, we obtained the best results by utilizing a combination of uni-grams and bi-grams, together with POS tagging. With respect to the traditional machine learning classifiers, and for example SVM, the best performance was achieved with the use of radial basis function kernel, loss of 0.12, seed of 1, and without normalization of input data.

For each of the datasets shown in Table 2, we split the data randomly as 75% to train and 25% to test the proposed sentence classifier system. The best accuracy results using our proposed predictive model were obtained by MWF=2%, EP=25, WS=2, ITR=10 configuration. Regarding the BoW features set, we utilized a combination of uni-grams, bi-grams, and POS tagging. Using the proposed predictive model, the vocabulary size for ADEs#1_Combined, ADEs#2_Combined, and ADEs#3_Combined datasets were 15125, 26,448, and 37,524, respectively.

Whereas with the use of BoW, when it utilized uni-grams, bi-grams, and POS, the vocabulary size was 33,567, 59,941, and 76,758 for datasets ADEs#1_Combined, ADEs#2_Combined, and ADEs#3_Combined. For SVM, decision tree, and naïve Bayes classifiers, we utilized Weka library (version 3.7.12) [66] running on hadoop-2.7 [67] by the use of Hadoop distributed file system.

**Figure 4:**
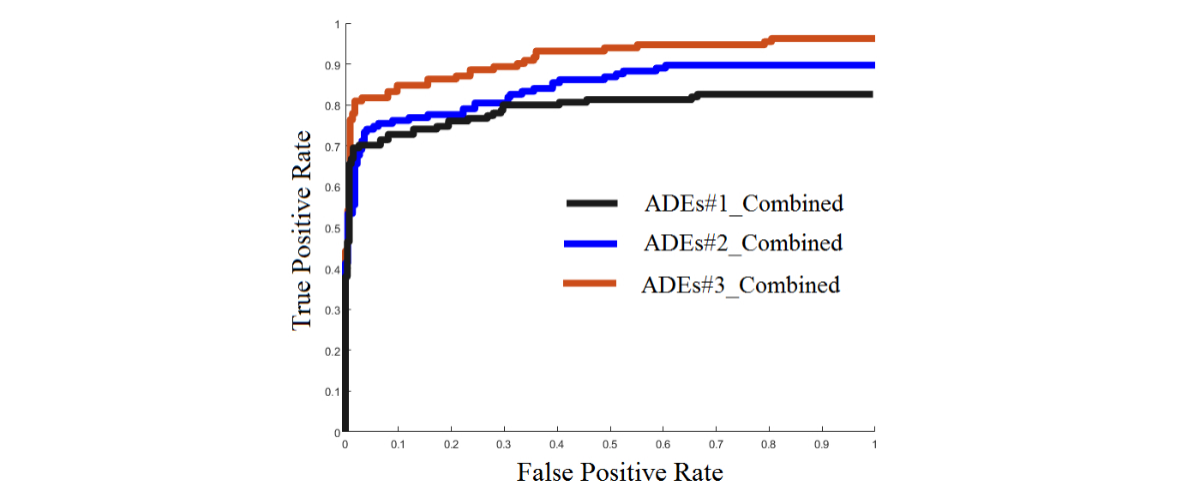
This figure shows the area under the curve (AUC) of our proposed predictive model. ADEs: adverse drug events.

The study shows that bigNN system generates better results in comparison with traditional BoW along with SVM, decision tree, and naïve Bayes classification algorithms. Area under the curve (AUC) of our proposed predictive model across all three datasets was also analyzed, and it is shown in Figure 4.

The current test results are only as good as the predictive model developed in the training phase. We accomplished further experiments just to make sure that the predictive model is sufficiently accurate in assigning appropriate classes for new unlabeled data records. Our approach was to rely on human reviews. We fed the proposed word2vec predictive model with new unlabeled data records and gave a random subset of the system output five hundred system-labeled instances to two domain experts to review. We got 87.6%, 86.1%, and 88.7% in average for accuracy, precision, and recall, respectively.

We briefly summarized the experimental results as follows:

The results we obtained showed that the use of combined dataset was better than the use of either source individually (Table 1) for all the models and is statistically significant (at *P*=.04).

The results illustrated in the Multimedia Appendix 2 show that a greater *epoch* along with a greater *iteration* with the use of *window size* of two tend to be useful over all datasets using bigNN system, and the result is statistically significant (at *P*=.02). However, it requires a longer training time.

The comparative study shown in Table 2 demonstrates that the bigNN system was able to generate better results in comparison with traditional BoW along with SVM, decision tree, and naïve Bayes classification algorithms. Performing a *t* test on AUC matched by those models shows statistically significant differences (at *P*=.03) between our bigNN system and those two models utilizing BoW along with decision tree and naïve Bayes. It also shows no statistically differences employing BoW and SVM.

**Table 2:** The comparisons of our big data neutral network (bigNN) system with traditional bag-of-words (BoW) method using support vector machine (SVM), decision tree, and naïve Bayes classifiers.

Dataset ID	Learning method	Number of sentences	Accuracy (%)	Precision (%)	Recall (%)	Area under the receiver operating characteristic	Training time (min)
ADEs#1_Combined^a^	bigNN^b^ system	7360	88.7	88.5	89.4	0.842	45.7
ADEs#1_Combined	BoW^c^ + SVM^d^	7360	89.4	88.3	88.0	0.841	66.3
ADEs#1_Combined	BoW + decision tree	7360	84.0	83.7	82.1	0.775	49.5
ADEs#1_Combined	BoW + naïve Bayes	7360	83.7	82.1	83.5	0.763	48.9
ADEs#2_Combined	bigNN system	14,017	89.1	88.9	89.3	0.874	69.5
ADEs#2_Combined	BoW + SVM	14,017	89.5	88.0	89.7	0.875	88.9
ADEs#2_Combined	BoW + decision tree	14,017	85.5	84.9	84.5	0.861	75.2
ADEs#2_Combined	BoW + naïve Bayes	14,017	84.3	84.0	85.7	0.855	73.8
ADEs#3_Combined	bigNN system	21,843	92.7	93.6	93.0	0.905	121.7
ADEs#3_Combined	BoW + SVM	21,843	92.5	94.0	93.2	0.911	159.5
ADEs#3_Combined	BoW + decision tree	21,843	88.3	87.5	87.2	0.868	131.5
ADEs#3_Combined	BoW + naïve Bayes	21,843	87.5	86.2	85.8	0.851	135.3

^a^ADEs: adverse drug events.

^b^bigNN: big data neutral network.

^c^BoW: bag-of-words.

^d^SVM: support vector machine.

Table 2 also shows that our bigNN system was faster than the BoW method along with SVM, decision tree, and naïve Bayes classification algorithms. Performing a *t* test on training time matched by those methods presents statistically significant differences (at *P*=.03) between our proposed model and all those three models developed by BoW along with SVM, decision tree, and naïve Bayes.

The AUCs presented in Figure 4 show that larger training samples tend to add benefit when making an accurate and reliable predictive model.

To further analyze the results, the next section will present a set of ADEs’ scientific visualizations obtained using the proposed framework.

### Scientific Visualization of ADEs

Scientific visualization is concerned with representing large and highly dimensional information by means of charts, graphs, and images. The general objective of any scientific visualization is to improve understanding of the data being investigated. In this section, we scientifically visualize the ADE information extracted by our proposed system. Once the system was trained across the different datasets, we fed the system new unlabeled data. This included 92,681,359 sentences from biomedical articles downloaded from PubMed Central and 1,624,117 sentences from social media, including MedHelp, Patient, and WebMD. There are considerable amounts of drugs and adverse events, and it is beyond the scope of this paper to visualize all of them. Here, we limited ourselves to the list of 28 drugs as illustrated in Table 3. The proposed system could find 12,265 ADE sentences from biomedical articles and 181 ADE sentences from three social media sources using the drug list defined.

**Figure 5:**
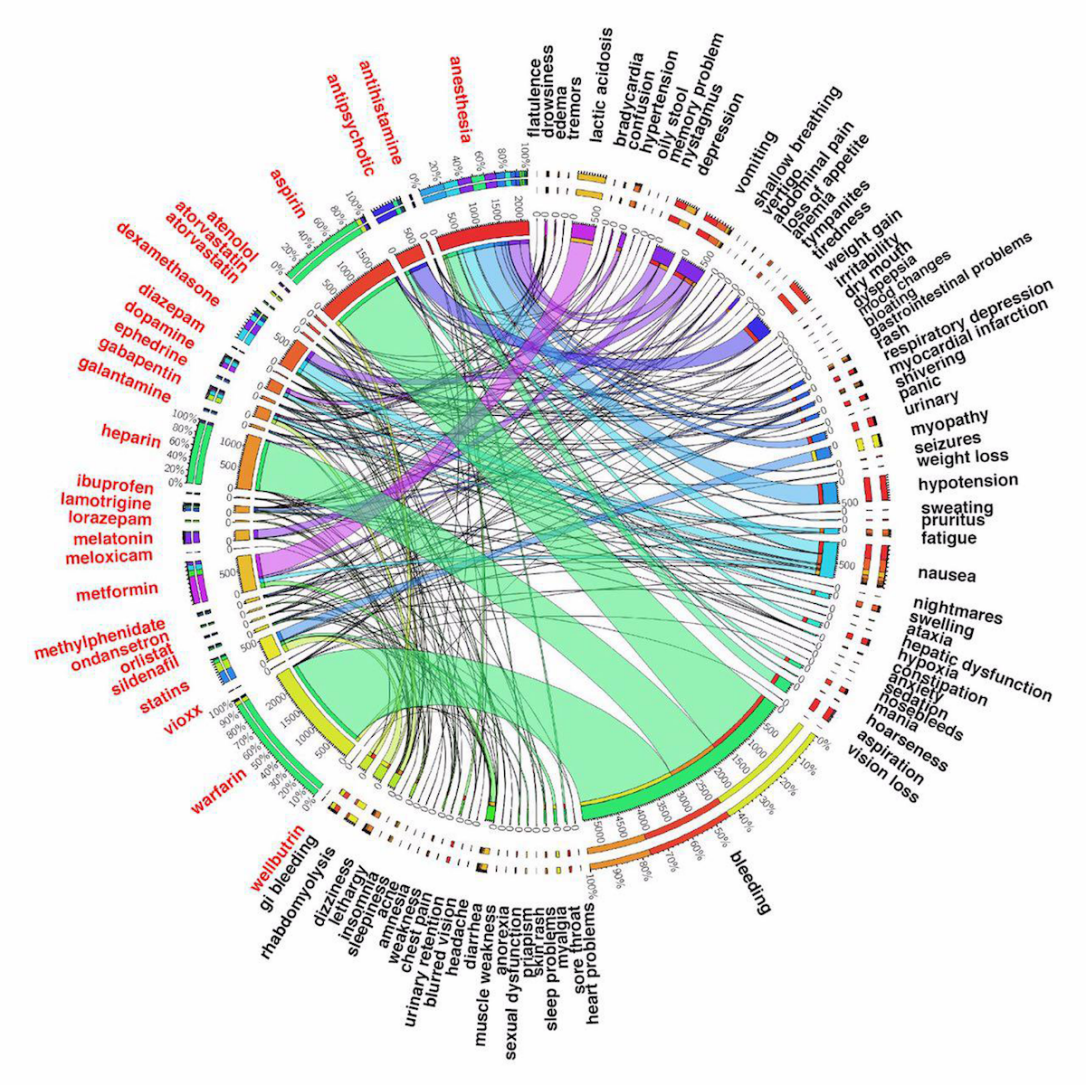
The adverse drug events’ (ADEs’) visualization results obtained from biomedical articles. One can see the number of “anesthesia” observations is 2186, where the most frequent adverse drug events are “hypotension,” “nausea,” “aspiration,” and “depression,” respectively. Using the CI of 95%, Pr(hypotension|anesthesia) is between 20.5% and 23.5%, and Pr(nausea|anesthesia) is some point between 12.0% and 14.7%. Another example is “gabapentin” where the most frequent adverse drug events based on ADE sentences extracted from biomedical articles are “dizziness,” “nausea,” “fatigue,” and “edema” whereas the number of “gabapentin” is 261. Using the CI of 95%, Pr(dizziness|gabapentin) is between 33.0% and 44.2%.

**Figure 6:**
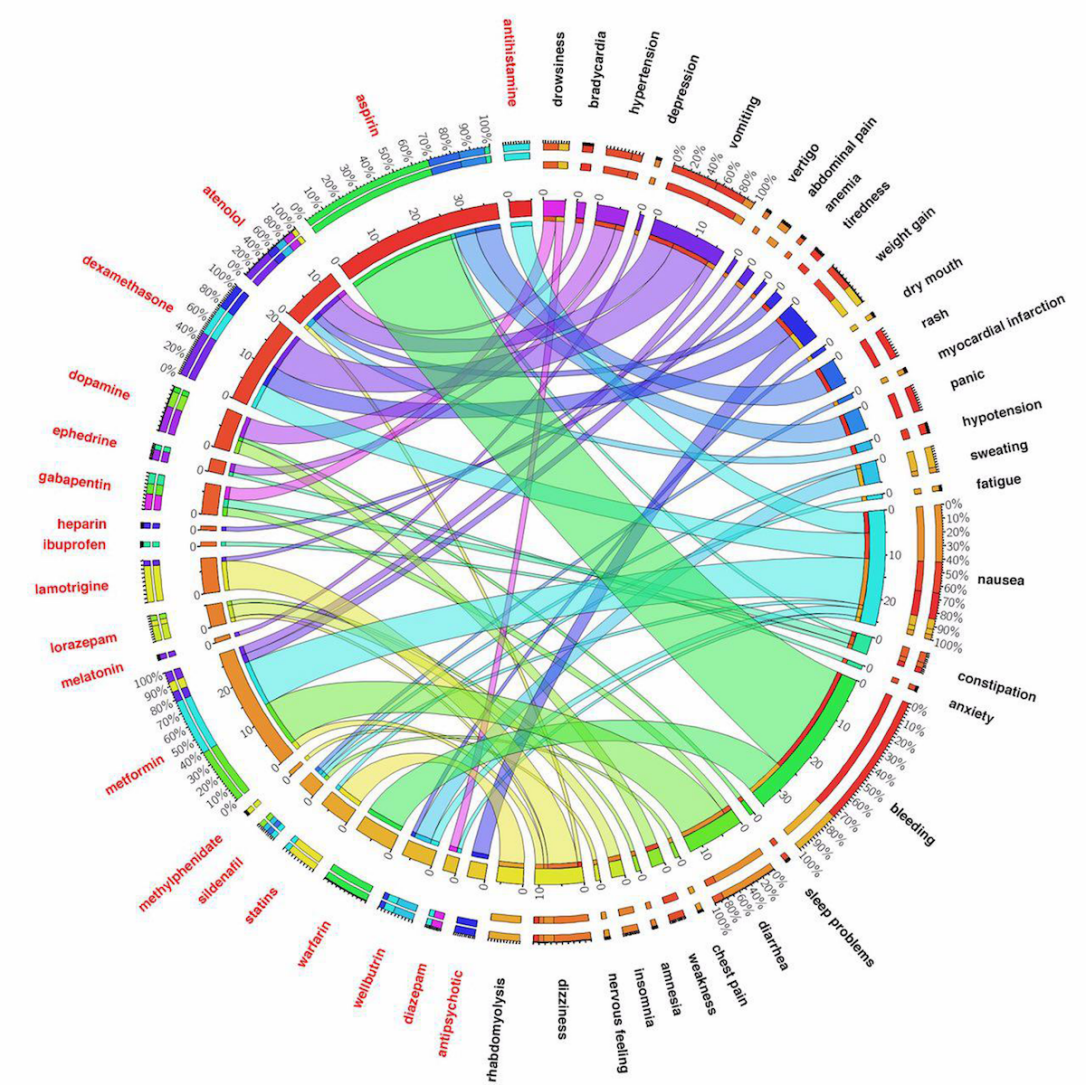
The adverse drug events’ (ADEs’) visualization results obtained from health-related social media. One can see the number of “metformin” observations is 28, where its most frequent ADEs are “nausea,” “diarrhea,” “vomiting,” “dizziness,” and “stomach pain,” respectively. Another example is “atenolol” and its most frequent adverse events are “depression,” “bradycardia,” “hypotension,” “tiredness,” and “dizziness.” From the adverse events aspect, this figure shows, for example, “nausea” as an adverse event is mostly associated with “metformin,” “dexamethasone,” “antihistamine,” “wellbutrin,” and “sildenafil”.

**Figure 7:**
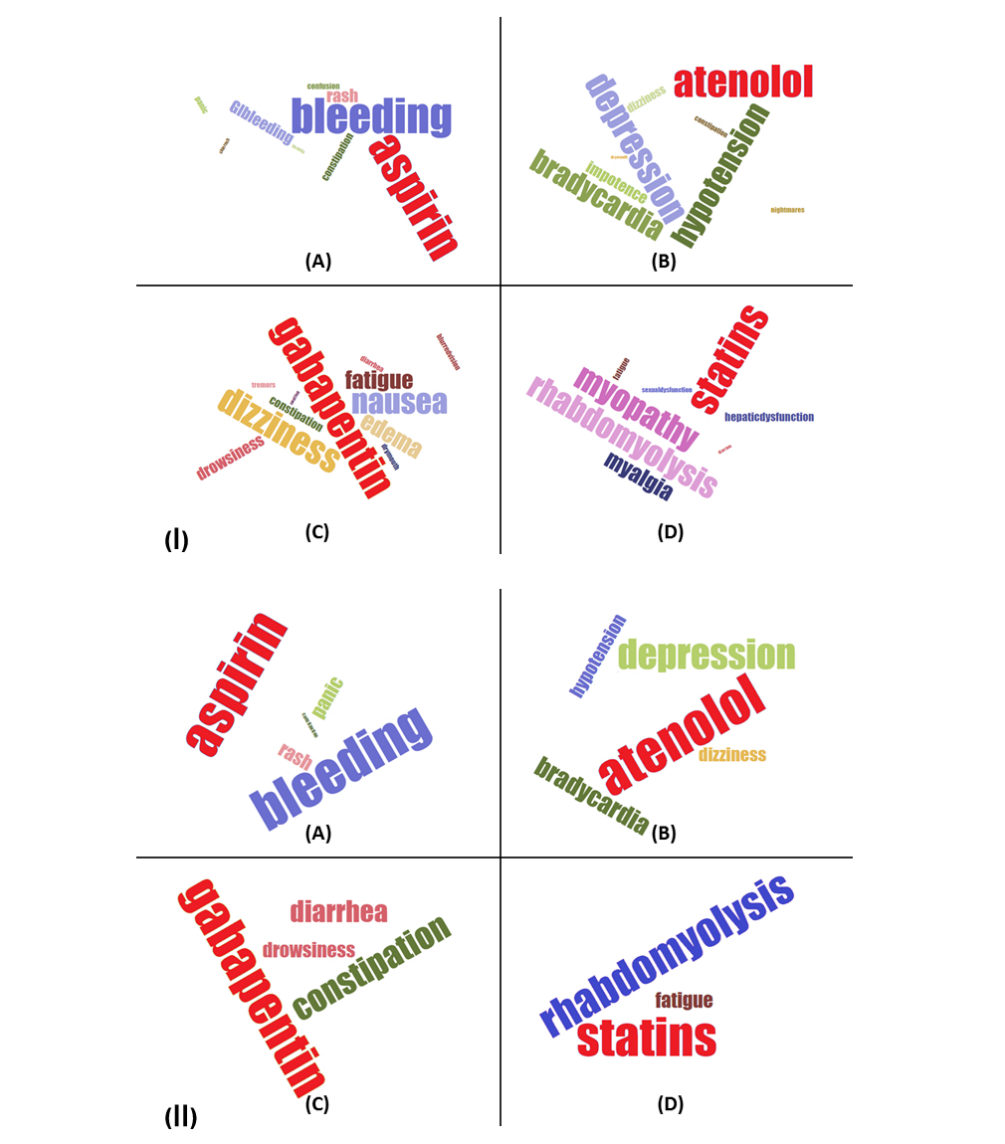
The word cloud representations for “aspirin,” “atenolol,” “gabapentin,” and “statins.” (1) These results are obtained from biomedical articles. Whereas the most frequent adverse dug events for “statins” are “myopathy,” “rhabdomyolysis,” “myalgia,” “fatigue,” and “hepatic dysfunction” as shown in (D), the most frequent ADEs for “atenolol” are “depression,” “hypotension,” “bradycardia,” and “impotence,” respectively (B). (2) These results are extracted from social media. Whereas the most frequent ADEs for “statins” are “rhabdomyolysis” and “fatigue” (D), the most frequent ADEs for “atenolol” are “depression,” “bradycardia,” “hypotension,” and “dizziness,” respectively (B).

Figures 5 and 6 show ADE discovery visualization results obtained from the biomedical articles and social media, respectively. Figure 7 represents a set of word cloud examples generated for ADEs associated with “aspirin,” “atenolol,” “gabapentin,” and “statins.” A word cloud is a graphical representation composed of different words contained in a corpus, in which the size of every word indicates its frequency or importance to the text.

**Table 3:** The list of the drugs used to make the scientific visualization results.

Row ID	Drug name
1	Anesthesia
2	Antihistamine
3	Antipsychotic
4	Aspirin
5	Atenolol
6	Atorvastatin
7	Azithromycin
8	Dexamethasone
9	Diazepam
10	Dopamine
11	Ephedrine
12	Gabapentin
13	Galantamine
14	Heparin
15	Ibuprofen
16	Lamotrigine
17	Lorazepam
18	Melatonin
19	Meloxicam
20	Metformin
21	Methylphenidate
22	Ondansetron
23	Orlistat
24	Sildenafil
25	Statins
26	Vioxx
27	Warfarin
28	Wellbutrin

## Discussion

### Principal Findings

Modern medical data sources ranging from clinical trials, EHRs, and medical case reports to scientific articles and patients’ blog posts are rapidly growing in size and complexity, and scientific biomedical articles, as well as health-related social media are under-researched data sources for biomedical studies. Thus, there is a pressing need to develop efficient solutions to harness this wealth of data using advanced computational methods such as artificial intelligence and big data machine learning.

With this contribution, an attempt was made to design and develop a computational bigNN system to detect, analyze, and visualize ADEs from massive data sources obtained from PubMed Central, a widely referenced repository of scientific articles, and the social media blog posts existing in MedHelp, Patient, and WebMD. The model was developed using the word2vec neural network architectural on top of the Apache Hadoop cluster, Apache Spark, and Elasticsearch No-SQL distributed database to tackle efficient big data ADE identification. We accomplished extensive experimental validations to ensure that the proposed predictive model can accurately assign appropriate classes (eg, ADEs or No-ADEs) for both current and also new unlabeled data records. Our trained system was able to detect a number of well-known ADEs from unlabeled data taken directly from the literature. A list of well-known ADEs can be found in Multimedia Appendix 1. Warfarin is an extremely effective anticlotting agent primarily used for patients at high risk for stroke or heart attack because of atrial fibrillation that, up until recently, has been a cornerstone of treatment. One common and potentially serious adverse effect of warfarin therapy is bleeding. This can occur because of changes in diet, drug interactions, or spurious physiological changes. The effectiveness of warfarin coupled with a high risk for serious bleeding events led to extensive research and publication in the medical literature, which is very apparent in our data visualization. Similarly, the widespread use of aspirin, which also carries a risk of bleeding, has been extensively studied for primary prevention of heart attack, colorectal cancer, and for secondary preventions of cardiovascular events, which is also captured in our results. Our system also identifies serious but rare side effects such as lactic acidosis caused by metformin use and rhabdomyolysis attributed to statin therapy. Common drug side effects are also captured, although with a smaller number of hits. Examples in Figure 5 include ADE pairs of metformin and diarrhea, antipsychotics and weight gain, gabapentin and dizziness, and drowsiness with antihistamines.

The findings of our system shed light on areas for future work and on inherent challenges with semantics and context in NLP. The following examples will illustrate some of these challenges. Our system identifies nausea and vomiting as ADEs associated with dexamethasone. Although it is true that dexamethasone can cause these reactions, it is commonly prescribed to prevent nausea and vomiting associated with chemotherapy. Without more contextual clues from the free text in the articles, it is impossible for us to decide whether dexamethasone is being identified as a treatment or a causal agent in this case. Another example where our system identified an indication as an ADE was with lamotrigine and seizures. Seizure is a primary indication for the use of lamotrigine and not a causal agent. Successfully classifying these edge cases may require additional labeled data, a larger window size, or other unexplored techniques and is an area for further study.

The ADEs identified from our analysis of social media provide a number of interesting similarities and differences with those in the literature. In our social media results, as might be expected, we see a larger proportion of ADEs related to the more common side effects of drugs as compared with the literature. For example, we see a large proportion of sentences identified for nausea and diarrhea with the use of metformin and fewer mentions of abdominal pain and vomiting. This parallels nicely with the incidence expected in real-world use. In contrast, we see a high proportion of sentences labeled for lactic acidosis, an extremely rare ADE associated with metformin use from the literature. The number of sentences describing adverse drug events in biomedical text articles is highly variable and includes influencing factors such as the severity of the drug reaction, safety concerns eliciting directed study, and the goals and intent of the research paper. Non-life-threatening ADEs are less important to clinical researchers, assuming they do not result in discontinuation, compared with serious reactions. In a similar way, side effects reported in social media will naturally include more common side effects, particularly because they are impactful to the patient taking the medication. In future work, text mining should take advantage of these naturally occurring differences. Publications in the biomedical literature or postings in social media, especially early after the release of a novel drug, may include case studies or reports of side effects not seen in clinical trials that could be detected by our system before the signal reaches the critical detection threshold of reporting systems such as the FDA Adverse Event Reporting System.

### Limitations

We acknowledge some limitations to this research study. Assessing the quality of scientific journals is a difficult task but important for narrowing the search space of candidate articles. In this work, we attempt to combine three ranking indices in the hope of identifying journals with the most credible information without generating a hand-curated list. It may be the case that our approach excludes journals that would be extremely useful in identifying ADEs but are excluded based on a low combined score. In addition, some journals will provide a richer source of information on ADEs than others based on their intended audience and subject matter irrespective of any ranking criterion. This leaves the question of which journals to focus on for ADE text mining open for further exploration. In the scientific articles, as well as social media blog posts, we noticed that different people may use different terms to discuss a similar single adverse event. For example, the terms “mood changes” and “mood swings” are often used interchangeably, equally meaning “mood changes.” A robust dictionary-based methodology may help address this issue. Additionally, text mining of the social media comments posted by patients is a really challenging task, as the comments are often written in an informal way. As we have a smaller number of labeled sentences from this source, we didn’t address overcoming the differences in phrasing, spelling errors, or other problems introduces from these posts. No fuzzy matching or specialized dictionaries were used on either source, so if there are spelling errors in the drug name, adverse event, or indication, the sentence would have been excluded from evaluation. Furthermore, our proposed big data neural network model is more appropriate for the short-length text data (eg, a sentence) classification rather than the long-length text data (eg, full text articles) categorization. Advanced tokenization systems, and in particular, a medical literature–based tokenization system will be useful with short-length text data.

### Conclusions

The present contribution utilized a bigNN system to discover only ADEs; however, there are several interesting applications to leverage the proposed system. For example, the proposed big data analytics pipeline could help study drug repurposing and impact drug development, particularly in analyzing the success rates of new medications. The social media demographic information (eg, age, gender, ethnicity, and location) supports use of the current contribution to explore ADEs and drug indications discussed by different demographic groups. For future work, we intend to further explore the application of the proposed framework to medical informatics, and particularly drug analyses, extending the work for social media–based ADE discovery discussed by different demographics groups. We would enhance the proposed framework to tackle the problem of semisupervised learning with multiple labels, rather than only a single label. We also plan to make a sentence-based ADE discovery dataset and present it publicly and make it freely available to the research community.

## Appendix

Multimedia Appendix 1 Selecting the relevant documents, Building the training set, Word2vec neural network model, Well-known ADEs.

Multimedia Appendix 2 bigNN experimental validations.

## Abbreviations

ADE: adverse drug event
AUC: area under the curve
BigNN: big data neutral network
BoW: bag-of-words
EHR: electronic health record
EP: epoch
ITR: iteration
MWF: minimum word frequency
NLP: natural language processing
POS: part-of-speech
SVM: support vector machine
WS: window size
XML: extensible markup language
