# Supplementary material for: Adverse Drug Event Discovery Using Biomedical Literature: A Big Data Neural Network Adventure
Source: JMIR Med Inform. 2017 Dec 8;5(4):e51. doi: 10.2196/medinform.9170 (PMC5741828; doi:10.2196/medinform.9170)
Supplement: Multimedia Appendix 1 [file medinform_v5i4e51_app1.pdf]

Appendix A

A.1 How we select the relevant documents

For scientific journals, we combine three journal ranking indices: (1) Impact Factor [11-12], (2) Eigenfactor [13-14], and (3) SJR (Scientific Journal Ranking) [15-16]. The scoring function below is employed to calculate the score of each article.

$$\textit{Score (articles)} = \textit{Impact Factor (journal)} + \textit{Eigenfactor (journal)} + \textit{SJR (journal)} \qquad (1)$$

Each individual ranking index above comes in a different range. The journals' Impact Factors for the articles we downloaded from PubMed range from 137 to 0, Eigenfactors and SJR vary from 1.813 to 0, and from 9.92 to 0 respectively. We first normalize (Equation 2) to adjust the values measured on different scales to a common scale of 0-1 range.

$$z_i = \frac{x_i - \min(x)}{\max(x) - \min(x)} \qquad (2)$$

Next, we find the average value of every scale individually, and calculate the average score as:

$$\textit{avg (score)} = \textit{avg (Impact Factor)} + \textit{avg (Eigenfactor)} + \textit{avg (SJR)} \qquad (3)$$

Once we calculate the average scores, we select only the articles where an associated journal's score that is above average. Examples of journals with above average scores include Genome Biology [17], Journal of Clinical Oncology [18] and Nature Chemical Biology [19] to name just a few. Regarding social media, we use only those that are sound and well-founded in health-related domains, such as MedHelp [20], Patient [21], and WebMD [22].

A.2 How we built the training set

Figure A.1 shows the web application we developed so that the domain experts (three domain experts) could vote for individual sentences as ADEs, No-ADEs, or Not Decided. Two examples of the ADEs sentences include: "*Gastrointestinal bleeding is one of the most frequent complications of warfarin and can occur when the warfarin level exceeds the target range; the warfarin level can fluctuate owing to dietary factors, other medications, or some genetic factors*", and "*During this study a total of seven gabapentin-treated patients withdrew*

from the study due to adverse events which included dizziness, abdominal pain, body odor, headache, diarrhea, abnormal thinking, nausea, and confusion". Examples of No-ADEs sentences include: "*Based on the different course of disease, many terms have been used to describe sclerosing mesenteritis, including mesenteric lipodystrophia, retractile or liposclerotic mesenteritis, mesenteric Weber-Christian disease, and xantogranulomatous mesenteritis*", and "*Statins are a class of drugs often prescribed by doctors to help lower cholesterol levels in the blood*".

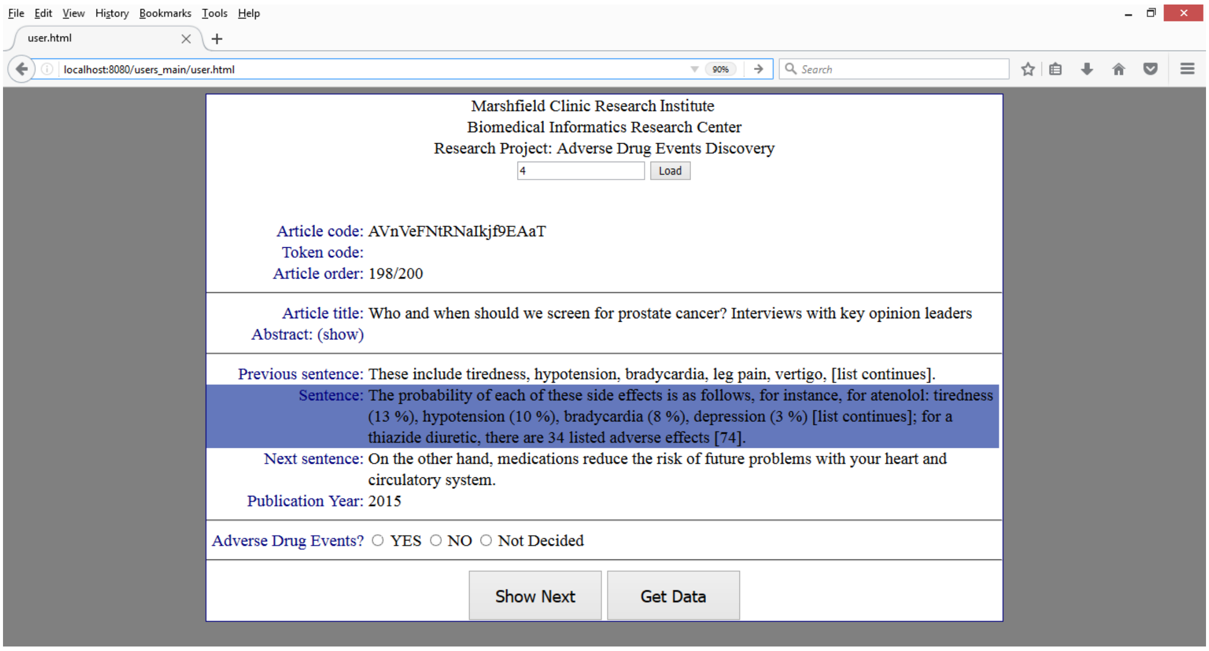

Figure A.1. A snapshot of the web application developed to assist our domain experts to accomplish manual annotation. The domain experts were able to see article title, its abstract, previous and next sentences, and also the publication year in addition to the sentence needed to be selected as ADEs, No-ADEs, or Not Decided. Finally, we were interested in only two different classes: (1) ADEs, and (2) No-ADEs to solve a binary classification problem.

Before the large scale of manual extraction, we have performed the evaluation of inter-rater reliability (IRR) among the three domain experts. Based on two hundred sentences evaluated by our three domain experts, the IRR was reported as 0.84 as Kappa statistics [25-26], which indicates very good concordance and agreement of our domain experts.

Totally, we have generated 7,360 human-labeled sentences to train our model which includes 6,960 biomedical articles and 400 social media posts. It took three domain experts in average two weeks to manually annotate all the sentences.

A.3 Word2vec neural network model

The word2vec algorithm is an important technique used in neural network language modeling and it is categorized in two different learning strategies: (1) Continuous bag-of-words (CBOW), and (2) Skip-gram. CBOW predicts a target word given a context, and skip-gram learning strategy predicts a target context given a word [1-2]. The two learning strategies of word2vec model are originally shallow neural networks; however, the representations acquired from these models can be used in various applications of deep learning.

Using the skip-gram learning algorithm, the target word is at the input layer and the context words will be on the output layer. In our proposed method, we developed an extended version of word2vec namely sentence2vec, employing the skip-gram model which is able to produce more accurate results on large-scale datasets [3]. Before delving into the detail of word2vec skip-gram and sentence2vec models, we shall explain the vector representation of word2vec for words across a corpus. Figure A.2 shows an example of word2vec vector representation for five words, including W1 to W5 amongst three sentences. Window Size is one of the Word2vec internal parameters that defines the context window, and in this example, we utilized a Window Size of 2, meaning that the vector of word “W1” is directly affected by the words “W2” and “W5”, and “W2” can be directly affected by four more words “W1”, “W3” “W4” and “W5”. In a very similar way, the vector of “W5’’ will be affected by the words “W1”, “W2’’ and “W4” as shown in Figure A.2.

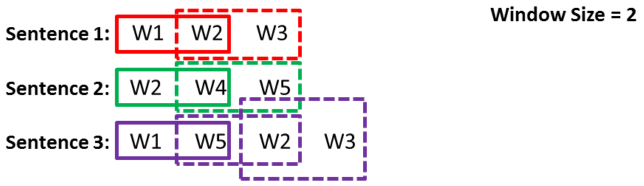

(A)

	W1	W2	W3	W4	W5
W1	0	1	0	0	1
W2	1	0	1	1	1
W3	0	1	0	0	0
W4	0	1	0	0	1
W5	1	1	0	1	0

(B)

Figure A.2. This figure illustrates word2vec representation vector for five distinct words across three different sentences. (A) Shows three sentences including some words, and a sliding window with size 2 is used to

generate word2vec representation vector. (B) Presents word2vec representation vector for every word in the corpus. For instance, the word2vec vector for W1 and W2 are 0 1 0 0 1 and 1 0 1 1 1 respectively. Using CBOW, we are predicting P(W(t)|W(t-1),W(t+1)) and for Skip-gram P(W(t-1),W(t+1)|W(t)).

Using skip-gram as a feedforward neural network, words are read into the vector one at a time, and scanned back and forth within a certain range as N-grams. N-gram is a contiguous sequence of N terms from a given sentence [4-5], and it is likely the N-th model of uni-gram, bi-gram, tri-gram, or four-gram. The N-gram is then fed into a neural network to account the significance of a given word vector. Skip-gram is able to predict the surrounding words given the current word, and it has the training complexity architecture as follows:

$$Q=C\times(D+D\times\log_2(V)) \qquad (4)$$

where the maximum distance for the words is C, D are word representations, and V is the dimensionality. Thus, for each training word, we will select randomly a number R in range $<1;C>$ and employ R words from history, and R words from the future of the chosen word as correct labels. This requires us to do $R\times2$ word classifications with the chosen word as input and each of the R+R words as output. Utilizing the binary tree representations of the vocabulary, the number of output units that require evaluation could come down to approximately $\log_2(V)$ [1, 2, 6]. The skip-gram neural network model is shown in Figure A.3. We use v_{W_I} to define the input vector of the only word on the input layer. The weights between the input layer and the output layer could be represented using a $V\times N$ matrix W in which each row of W is the N-dimension vector representation v_W of the related word of the input layer. The number of input and output nodes is equal to the vocabulary size, and the number of hidden nodes ranges from 1 to the maximum number of the vocabulary size. Row i of W is v_w^T, and given a word (context) along with two assumptions as $x_k=1$ and $x_{k'}=0$ for $k'\neq k$, we will have the definition of the hidden layer outputs h as equation (5) such that it copies the k-th row of W to h. v_{w_I} is the vector representation of the input word w_I, and it shows that the activation function of the hidden layer units is linear [2, 6].

$$h=W^Tx=W^T_{(k,.)}:=v^T_{w_I} \qquad (5)$$

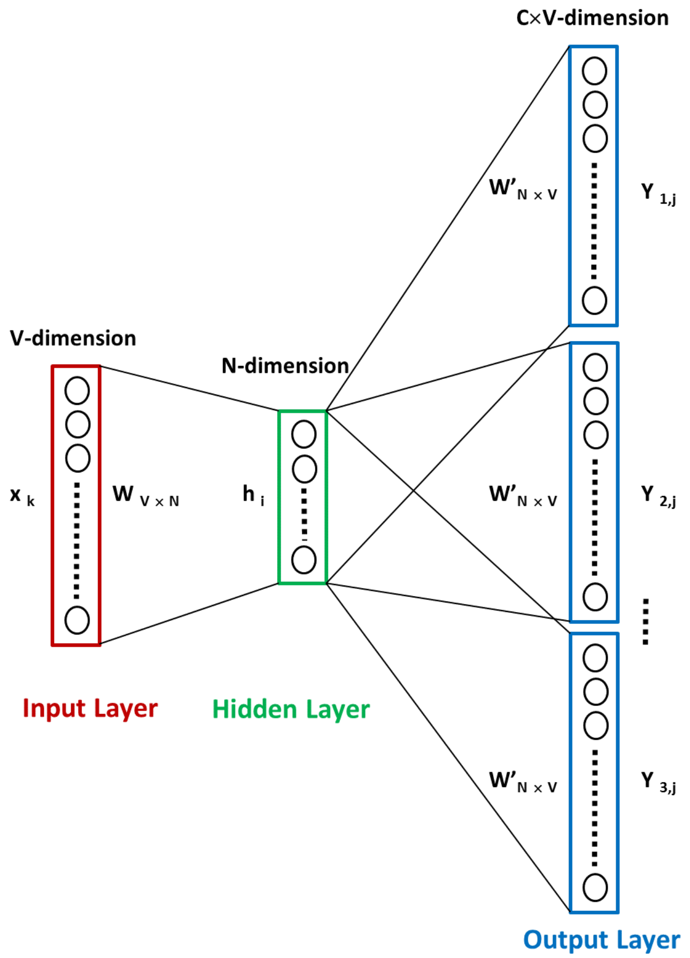

Figure A.3. The word2vec skip-gram learning model. V indicates the vocabulary size, and C refers to the context size.

Here, from the hidden layer to the output layer, there will be a different weight matrix W' which is an $N{\times}V$ matrix as W'_{ij}. Employing the entire weights, the score u_j for every word in the context could be estimated as:

$$u_j = v'^{T}_{wj} h \tag{6}$$

where v'_{wj} is the j-th column of the matrix W'. In almost all classification problems we take advantage of a condition that the classes are mutually exclusive. Considering an activation function (e.g., Hard limit, Saturating linear, Log-sigmoid) in the output layer, an adequate neural network architecture for such requirement is Max-layer output in which it could

generate probability of 1 for the maximum output of the previous layer and probability of 0 for the rest of the output nodes. The problem is that, such output layer could not be differentiable and therefore it will be very challenging to train. Utilizing “Softmax” function as an activation function in the output layer, works nearly similar to Max-layer and it could be differentiable if trained by the gradient descent [7-10]. The Softmax function will increase the probability of maximum value of the previous layer compared to other value [1, 2, 6]. Hence, for the discussed neural network architecture, the “Softmax” function is used as an activation function in the output layer. In the output layer, every output is calculated using the same hidden $\rightarrow$ output matrix as equation (7).

$$p(W_{cj}=W_{Oc}|W_I)=y_{cj}=\frac{\exp(u_{cj})}{\sum_{j'=1}^{V}\exp(u_{j'})} \qquad (7)$$

where $w_{c,j}$ is the j-th word on the c-th panel of the output layer, $w_{O,c}$ is the exact c-th word in the output context words, w_I is the input word, $y_{c,j}$ is the output of the j-th unit on the c-th panel of the output layer, and $u_{c,j}$ is the neural network input of the j-th unit on the c-th panel of the output layer. Since the output layer panels are sharing the same weights together, therefore:

$$u_{cj} = u_j = v'^{T}_{wj} h, \text{ for } c = 1, 2, 3, \ldots, C \qquad (8)$$

where V'_{w_j} is the output vector of the j-th word in the vocabulary, and w_j as well as V'_{w_j} are taken from a column of the hidden $\rightarrow$ output weight matrix W'. To train the neural network model, we utilized a training set and applied forward pass to check the error first, and then we employed back propagation to find out the optimal weights across the network.

To further explain the word2vec skip-gram neural network architecture, a kind of comprehensive view of the Figure A.3 assuming $V(vocabulary size) = 5$ and $C(context size) = 3$ is shown in Figure A.4. Considering forward propagation, calculation of weights from input layer to hidden layer would be as follows:

$$\begin{bmatrix} h1 \\ h2 \\ h3 \end{bmatrix} = W^T x = \begin{bmatrix} W_{11} & W_{21} & W_{31} & W_{41} & W_{51} \\ W_{12} & W_{22} & W_{32} & W_{42} & W_{52} \\ W_{13} & W_{23} & W_{33} & W_{43} & W_{53} \end{bmatrix} \begin{bmatrix} x_1 \\ x_2 \\ x_3 \\ x_4 \\ x_5 \end{bmatrix} \quad (9)$$

The calculation of weights from hidden layer to output layer is also shown in equation (10).

$$\text{Net(O1j)}\ \{1 \le j \le 5\} = W'^T h = \begin{bmatrix} W'_{11} & W'_{21} & W'_{31} \\ W'_{12} & W'_{22} & W'_{32} \\ W'_{13} & W'_{23} & W'_{33} \\ W'_{14} & W'_{24} & W'_{34} \\ W'_{15} & W'_{25} & W'_{35} \end{bmatrix} \begin{bmatrix} h_1 \\ h_2 \\ h_3 \end{bmatrix} \quad (10)$$

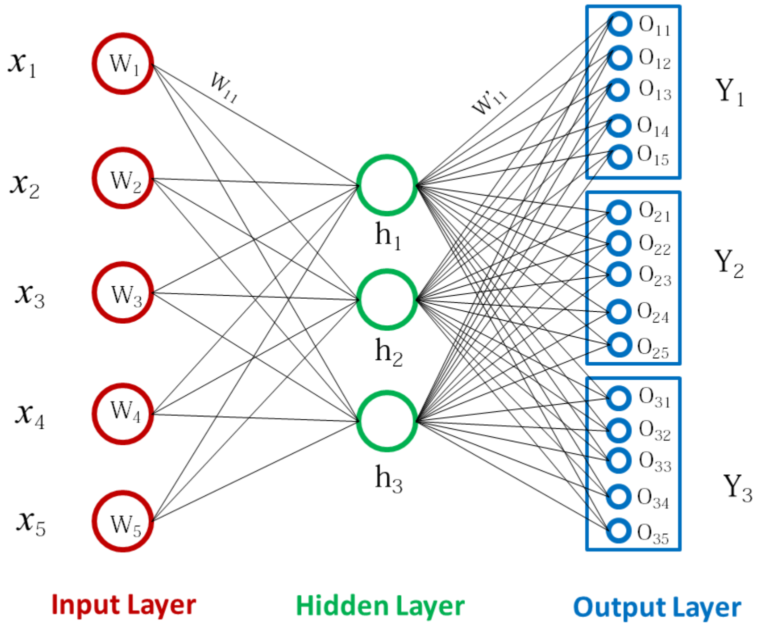

Figure A.4. A magnified view of word2vec skip-gram learning model with five input nodes, three hidden nodes, and three nodes creating fifteen outputs.

The calculation of the Softmax output for Y_{11}, Y_{12}, and Y_{13} would be equal to equations (11), (12), and (13) respectively. Similarly, we can calculate the Softmax output for Y_{14}, Y_{15}.

$$Y_{11} = \frac{e^{Net(O_{11})}}{e^{Net(O_{11})}+e^{Net(O_{12})}+e^{Net(O_{13})}+e^{Net(O_{14})}+e^{Net(O_{15})}} \quad (11)$$

$$Y_{12} = \frac{e^{Net(O_{12})}}{e^{Net(O_{11})}+e^{Net(O_{12})}+e^{Net(O_{13})}+e^{Net(O_{14})}+e^{Net(O_{15})}} \quad (12)$$

$$Y_{13} = \frac{e^{Net(O_{13})}}{e^{Net(O_{11})}+e^{Net(O_{12})}+e^{Net(O_{13})}+e^{Net(O_{14})}+e^{Net(O_{15})}} \quad (13)$$

As we discussed earlier, we are interested in minimizing the error. Therefore, and in this example (Figure A.4), the loss function would be defined by a conditional probability as equation (14).

$$\mathrm{E} = -\log p(W_{1j}, W_{2j}, W_{3j} \,|W_I) = -\log \prod_{c=1}^{3} \frac{e^{Net(O_{cj})}}{e^{Net(O_{11})}+e^{Net(O_{12})}+e^{Net(O_{13})}+e^{Net(O_{14})}+e^{Net(O_{15})}} \quad (14)$$

Generalizing the equation by considering the total context windows equal to C and vocabulary size as J:

$$E = -\log p(W_{1j}, W_{2j}, W_{3j}, \cdots, W_{Cj} \,|W_I) = -\log \prod_{c=1}^{C} \frac{e^{Net(O_{cj})}}{e^{Net(O_{11})} + e^{Net(O_{12})} + \cdots + e^{Net(O_{1J})}} \quad (15)$$

And we can make it short as:

$$E = -\log p(W_{1j}, W_{2j}, W_{3j}, \cdots, W_{cj} | W_I) = -\log \prod_{c=1}^{C} \left(\frac{e^{Net(O_{cj})}}{\sum_{j=1}^{J} e^{Net(O_{1j})}} \right) \quad (16)$$

By taking derivative with respect to $e^{Net(O_{cj})}$:

$$\frac{dE}{dNet(O_{cj})} = \frac{e^{Net(O_{cj})}}{\sum_{j=1}^{J} e^{Net(O_{1j})}} - Z_{cj} = Y_{cj} - Z_{cj} \quad (17)$$

where Y_{cj} is the outscore of j-th word of c-th context window. If j-th word of c-th context window is the actual output word, then Z_{cj}=1, otherwise Z_{cj}=0.

A customized version of the word2vec skip-gram algorithm, namely "sentence2vec", has been developed and used for the current study. In this model, the class of the sentence (e.g., ADEs or No-ADEs) was concatenated to the words list, so the model can make accurate guesses about a sentence's meaning based on past appearances in the corpus.

A.4 A list of well-known ADEs

Table A.1. Well-known ADEs used for system validation. This list was created using Micromedex available at https://www.micromedexsolutions.com on 8/31/2017.

Drug Name	Severity	ADE Category	ADE
Aspirin	Serious	Gastrointestinal	Gastrointestinal Ulcer
Aspirin	Serious	Hematologic	Hemorrhage
Aspirin	Serious	Ophthalmic	Exudative Age-Related Macular Degeneration
Aspirin	Serious	Otic	Tinnitus
Aspirin	Serious	Respiratory	Bronchospasm
Aspirin	Serious	Other	Angioedema
Aspirin	Serious	Other	Reye's syndrome
Lamotrigine	Common	Dermatologic	Rash (7% to 14%)
Lamotrigine	Common	Gastrointestinal	Abdominal pain (immediate-release, 5% to 10%)
Lamotrigine	Common	Gastrointestinal	Diarrhea (immediate-release, 6% to 11%; extended-release, 5%)
Lamotrigine	Common	Gastrointestinal	Indigestion (immediate-release, 2% to 7%)
Lamotrigine	Common	Gastrointestinal	Nausea (immediate-release, 7% to 25%; extended-release, 7%)
Lamotrigine	Common	Gastrointestinal	Vomiting (immediate-release, 5% to 20%; extended-release, 6%))

Lamotrigine	Common	Neurologic	Asthenia (immediate-release, 2% to 8%; extended-release, 6%)
Lamotrigine	Common	Neurologic	Ataxia (immediate-release, 2% to 11%)
Lamotrigine	Common	Neurologic	Coordination problem (immediate-release, 6% to 7%; extended-release, 3%)
Lamotrigine	Common	Neurologic	Dizziness (immediate-release, 7% to 54% ; extended release, 14%)
Lamotrigine	Common	Neurologic	Headache (immediate-release, 29%)
Lamotrigine	Common	Neurologic	Insomnia (immediate-release, 5% to 10%)
Lamotrigine	Common	Neurologic	Somnolence (immediate-release, 9% to 17%; extended-release, 5%)
Lamotrigine	Common	Neurologic	Tremor (immediate-release, 4% to 10%; extended-release, 6%)
Lamotrigine	Common	Neurologic	Vertigo (immediate-release, 2%; extended-release, 3%)
Lamotrigine	Common	Ophthalmic	Blurred vision (immediate-release, 11% to 25% (adults) and 4% (children); extended-release, 3%)
Lamotrigine	Common	Ophthalmic	Diplopia (immediate-release, 24% to 49% (adults) and 5% (children); extended-release, 5%)
Lamotrigine	Common	Psychiatric	Anxiety (immediate-release, 4%; extended-release, 3%)
Lamotrigine	Common	Psychiatric	Depression (immediate-release, 4%; extended-release, 3%)
Lamotrigine	Common	Reproductive	Dysmenorrhea (immediate-release, 5% to 7%)
Lamotrigine	Common	Respiratory	Rhinitis (immediate-release, 7% to 14%)
Lamotrigine	Common	Other	Pain (immediate-release, 5%)
Lamotrigine	Serious	Dermatologic	Erythema multiforme (less than 0.1%)
Lamotrigine	Serious	Dermatologic	Rash
Lamotrigine	Serious	Dermatologic	Serious (0.08% to 0.8%)
Lamotrigine	Serious	Dermatologic	Stevens-Johnson syndrome
Lamotrigine	Serious	Dermatologic	Toxic epidermal necrolysis
Lamotrigine	Serious	Hematologic	Anemia (immediate release, less than 0.1%)
Lamotrigine	Serious	Hematologic	Disseminated intravascular coagulation
Lamotrigine	Serious	Hematologic	Eosinophil count raised (immediate release, less than 0.1%)
Lamotrigine	Serious	Hematologic	Leukopenia (immediate release, 0.1% to 1%)
Lamotrigine	Serious	Hematologic	Thrombocytopenia (immediate release, less than 0.1%)
Lamotrigine	Serious	Hepatic	Liver failure
Lamotrigine	Serious	Immunologic	Drug reaction with eosinophilia and systemic symptoms
Lamotrigine	Serious	Neurologic	Aseptic meningitis
Lamotrigine	Serious	Other	Angioedema (less than 0.1%)
Lamotrigine	Serious	Other	Neuroleptic malignant syndrome
Metformin	Common	Endocrine Metabolic	Cobalamin deficiency (7% to 17.4%)
Metformin	Common	Gastrointestinal	Diarrhea (53.2% (immediate-release) ; 9.6% to 12.5% (extended-release))
Metformin	Common	Gastrointestinal	Flatulence (12.1%)
Metformin	Common	Gastrointestinal	Indigestion (7.1%)
Metformin	Common	Gastrointestinal	Malabsorption syndrome (up to 9.9%)
Metformin	Common	Gastrointestinal	Nausea (up to 25.5% (immediate-release); ; 6.7% (extended-release))
Metformin	Common	Gastrointestinal	Vomiting (up to 25.5%)
Metformin	Common	Neurologic	Asthenia (9.2%)
Metformin	Common	Neurologic	Headache (5.7%)
Metformin	Serious	Endocrine metabolic	Lactic acidosis
Metformin	Serious	Hepatic	Hepatitis
Warfarin	Common	Dermatologic	Alopecia
Warfarin	Serious	Cardiovascular	Cholesterol embolus syndrome
Warfarin	Serious	Cardiovascular	Tissue necrosis (Less than 0.1%)
Warfarin	Serious	Dermatologic	Calciphylaxis
Warfarin	Serious	Dermatologic	Tissue necrosis (Less than 0.1%)
Warfarin	Serious	Hematologic	Hemorrhage
Warfarin	Serious	Immunologic	Hypersensitivity reaction

Warfarin	Serious	Musculoskeletal	Compartment syndrome
Warfarin	Serious	Neurologic	Intracranial hemorrhage
Warfarin	Serious	Ophthalmic	Intraocular hemorrhage

References

1. Mikolov T, Chen K, Corrado G, Dean J. Efficient estimation of word representations in vector space. arXiv preprint arXiv:1301.3781. 2013 Jan 16.
2. Rong X. word2vec parameter learning explained. arXiv preprint arXiv:1411.2738. 2014 Nov 11.
3. Team DJ. Deeplearning4j: Open-source distributed deep learning for the JVM. Apache Software Foundation License. 2016;2.
4. Ordentlich E, Yang L, Feng A, Cnudde P, Grbovic M, Djuric N, Radosavljevic V, Owens G. Network-Efficient Distributed Word2vec Training System for Large Vocabularies. InProceedings of the 25th ACM International on Conference on Information and Knowledge Management 2016 Oct 24 (pp. 1139-1148). ACM.
5. Ju R, Zhou P, Li CH, Liu L. An Efficient Method for Document Categorization Based on Word2vec and Latent Semantic Analysis. InComputer and Information Technology; Ubiquitous Computing and Communications; Dependable, Autonomic and Secure Computing; Pervasive Intelligence and Computing (CIT/IUCC/DASC/PICOM), 2015 IEEE International Conference on 2015 Oct 26 (pp. 2276-2283). IEEE.
6. Goldberg Y, Levy O. word2vec Explained: deriving Mikolov et al.'s negative-sampling word-embedding method. arXiv preprint arXiv:1402.3722. 2014 Feb 15.
7. Mikolov T, Karafiát M, Burget L, Cernocký J, Khudanpur S. Recurrent neural network based language model. InInterspeech 2010 Sep 26 (Vol. 2, p. 3).
8. Chan W, Jaitly N, Le Q, Vinyals O. Listen, attend and spell: A neural network for large vocabulary conversational speech recognition. InAcoustics, Speech and Signal Processing (ICASSP), 2016 IEEE International Conference on 2016 Mar 20 (pp. 4960-4964). IEEE.
9. Snyman J. Practical mathematical optimization: an introduction to basic optimization theory and classical and new gradient-based algorithms. Springer Science & Business Media; 2005 Dec 15.
10. Burges C, Shaked T, Renshaw E, Lazier A, Deeds M, Hamilton N, Hullender G. Learning to rank using gradient descent. InProceedings of the 22nd international conference on Machine learning 2005 Aug 7 (pp. 89-96). ACM.
11. Amin M, Mabe M. Impact factors: use and abuse. International Journal of Environmental Science and Technology:(IJEST). 2004 Apr 1;1(1):1.
12. Kurmis AP. Understanding the limitations of the journal impact factor. JBJS. 2003 Dec 1;85(12):2449-54. **PMID: 14668520**

13. Bergstrom CT, West JD, Wiseman MA. The eigenfactor™ metrics. Journal of Neuroscience. 2008 Nov 5;28(45):11433-4. **PMID: 18987179**
14. Bergstrom CT, West JD. Assessing citations with the Eigenfactor™ metrics. Neurology. 2008 Dec 2;71(23):1850-1.
15. González-Pereira B, Guerrero-Bote VP, Moya-Anegón F. A new approach to the metric of journals' scientific prestige: The SJR indicator. Journal of informetrics. 2010 Jul 31;4(3):379-91.
16. Colledge L, de Moya-Anegón F, Guerrero-Bote V, López-Illescas C, El Aisati M, Moed HF. SJR and SNIP: two new journal metrics in Elsevier's Scopus. Serials. 2010 Nov 16;23(3):215-21.
17. Genome Biology, https://genomebiology.biomedcentral.com, Last accessed on Oct 09, 2017.
18. Journal of Clinical Oncology, http://ascopubs.org/journal/jco, Last accessed on Oct 09, 2017.
19. Nature Chemical Biology, http://www.nature.com/nchembio/index.html, Last accessed on Oct 09, 2017.
20. MedHelp. http://medhelp.org, Last accessed on Oct 02, 2017.
21. Patient. http://patient.info, Last accessed on June 25, 2017.
22. WebMD. http://www.webmd.com, Last accessed on Sep 18, 2017.
23. Apache Spark. https://spark.apache.org, Last accessed on June 19, 2017.
24. Elasticsearch. https://www.elastic.co, Last accessed on June 11, 2017.
25. Viera AJ, Garrett JM. Understanding interobserver agreement: the kappa statistic. Fam Med. 2005 May 1;37(5):360-3. **PMID: 15883903**
26. Di Eugenio B, Glass M. The kappa statistic: A second look. Computational linguistics. 2004 Mar;30(1):95-101.
