# Supplementary material for: Adverse Drug Event Discovery Using Biomedical Literature: A Big Data Neural Network Adventure
Source: JMIR Med Inform. 2017 Dec 8;5(4):e51. doi: 10.2196/medinform.9170 (PMC5741828; doi:10.2196/medinform.9170)
Supplement: Multimedia Appendix 2 [file medinform_v5i4e51_app2.pdf]

Appendix B

B.1 bigNN Experimental Validations

Table B. 1. Experimental analysis of the big data neutral network (bigNN) system. Training time shows only the elapsed time to train the model, and it does not reflect the time of the text-preprocessing tasks (eg, normalization). Minimum word frequency (MWF) allows ignoring all words in the vocabulary with total occurrences lower than MWF value. Epoch (EP) is the number of forward and backward passes of all training examples. Window size (WS) defines context windows size to generate a vector representation for words across the documents. Iteration (ITR) defines number of iterations done for each mini-batch during a training. These experiments were performed with the use of MWF=2%.

Dataset ID	Configurations	Number of sentences	Accuracy (%)	Precision (%)	Recall (%)	Training time (min)
ADEs#1_BA	EP[a]=5; WS[b]=1; ITR[c]=4	6960	81.5	81.3	80.4	21.7
ADEs#1_BA	EP=5; WS=1; ITR=10	6960	81.8	82.4	80.5	22.5
ADEs#1_BA	EP=25; WS=1; ITR=4	6960	84.8	85.0	84.2	34.4
ADEs#1_BA	EP=25; WS=1; ITR=10	6960	84.9	85.0	84.5	35.5
ADEs#1_BA	EP=5; WS=2; ITR=4	6960	81.8	82.0	82.4	22.6
ADEs#1_BA	EP=5; WS=2; ITR=10	6960	82.5	83.1	82.0	23.1
ADEs#1_BA	EP=25; WS=2; ITR=4	6960	84.3	84.7	85.6	36.4
ADEs#1_BA	EP=25; WS=2; ITR=10	6960	85.1	85.7	86.3	37.5
ADEs#1_SM	EP=5; WS=1; ITR=4	400	78.1	78.7	78.5	1.5
ADEs#1_SM	EP=5; WS=1; ITR=10	400	78.4	78.0	78.7	2.3
ADEs#1_SM	EP=25; WS=1; ITR=4	400	80.5	80.0	80.4	8.6
ADEs#1_SM	EP=25; WS=1; ITR=10	400	80.8	81.2	80.3	9.1
ADEs#1_SM	EP=5; WS=2; ITR=4	400	78.9	79.2	78.6	2.8
ADEs#1_SM	EP=5; WS=2; ITR=10	400	78.8	80.2	80.1	3.5
ADEs#1_SM	EP=25; WS=2; ITR=4	400	81.0	80.5	80.7	9.5
ADEs#1_SM	EP=25; WS=2; ITR=10	400	81.3	80.4	81.1	10.8
ADEs#1_Combined	EP=5; WS=1; ITR=4	7360	84.0	85.7	86.5	24.8
ADEs#1_Combined	EP=5; WS=1; ITR=10	7360	84.2	85.5	86.7	26.3
ADEs#1_Combined	EP=25; WS=1; ITR=4	7360	87.5	88.3	88.8	42.5
ADEs#1_Combined	EP=25; WS=1; ITR=10	7360	87.8	88.4	88.5	44.1
ADEs#1_Combined	EP=5; WS=2; ITR=4	7360	84.5	85.4	86.7	26.5
ADEs#1_Combined	EP=5; WS=2; ITR=10	7360	84.5	85.1	86.9	28.4

ADEs#1_Combined	EP=25; WS=2; ITR=4	7360	88.4	88.5	89.1	44.2
ADEs#1_Combined	EP=25; WS=2; ITR=10	7360	88.7	88.5	89.4	45.7
ADEs#2_BA	EP=5; WS=1; ITR=4	13,545	83.4	82.0	83.5	35.1
ADEs#2_BA	EP=5; WS=1; ITR=10	13,545	83.6	83.1	83.4	36.5
ADEs#2_BA	EP=25; WS=1; ITR=4	13,545	85.9	85.0	85.5	55.9
ADEs#2_BA	EP=25; WS=1; ITR=10	13,545	85.8	85.8	85.3	58.3
ADEs#2_BA	EP=5; WS=2; ITR=4	13,545	84.0	84.7	84.4	36.4
ADEs#2_BA	EP=5; WS=2; ITR=10	13,545	84.1	84.3	84.7	38.2
ADEs#2_BA	EP=25; WS=2; ITR=4	13,545	87.0	87.7	86.0	59.5
ADEs#2_BA	EP=25; WS=2; ITR=10	13,545	87.4	87.5	86.2	61.8
ADEs#2_SM	EP=5; WS=1; ITR=4	472	78.4	78.0	78.9	3.4
ADEs#2_SM	EP=5; WS=1; ITR=10	472	78.6	78.2	78.6	4.5
ADEs#2_SM	EP=25; WS=1; ITR=4	472	81.1	80.0	80.4	11.9
ADEs#2_SM	EP=25; WS=1; ITR=10	472	81.3	81.0	80.9	13.5
ADEs#2_SM	EP=5; WS=2; ITR=4	472	78.9	79.5	79.3	3.9
ADEs#2_SM	EP=5; WS=2; ITR=10	472	79.0	79.2	79.8	5.2
ADEs#2_SM	EP=25; WS=2; ITR=4	472	82.5	81.0	81.8	14.3
ADEs#2_SM	EP=25; WS=2; ITR=10	472	82.7	81.4	81.3	16.5
ADEs#2_Combined	EP=5; WS=1; ITR=4	14,017	85.1	86.5	86.9	39.2
ADEs#2_Combined	EP=5; WS=1; ITR=10	14,017	85.7	86.0	86.1	42.5
ADEs#2_Combined	EP=25; WS=1; ITR=4	14,017	88.0	88.9	89.1	65.8
ADEs#2_Combined	EP=25; WS=1; ITR=10	14,017	88.4	88.0	88.7	68.3
ADEs#2_Combined	EP=5; WS=2; ITR=4	14,017	85.3	85.0	87.7	39.8
ADEs#2_Combined	EP=5; WS=2; ITR=10	14,017	85.5	85.4	86.9	43.4
ADEs#2_Combined	EP=25; WS=2; ITR=4	14,017	89.0	89.3	88.5	67.1
ADEs#2_Combined	EP=25; WS=2; ITR=10	14,017	89.1	88.9	89.3	69.5
ADEs#3_BA	EP=5; WS=1; ITR=4	21,278	86.2	85.0	85.2	54.5
ADEs#3_BA	EP=5; WS=1; ITR=10	21,278	86.4	85.0	85.4	58.2
ADEs#3_BA	EP=25; WS=1; ITR=4	21,278	89.0	89.1	88.0	101.7
ADEs#3_BA	EP=25; WS=1; ITR=10	21,278	89.4	89.0	88.1	104.2
ADEs#3_BA	EP=5; WS=2; ITR=4	21,278	86.5	85.0	86.7	55.0
ADEs#3_BA	EP=5; WS=2; ITR=10	21,278	86.3	85.3	86.7	56.4
ADEs#3_BA	EP=25; WS=2; ITR=4	21,278	89.5	89.7	89.0	104.2
ADEs#3_BA	EP=25; WS=2; ITR=10	21,278	89.6	88.8	89.2	106.3
ADEs#3_SM	EP=5; WS=1; ITR=4	565	80.3	80.9	81.5	6.1

ADEs#3_SM	EP=5; WS=1; ITR=10	565	80.5	80.2	81.0	8.3
ADEs#3_SM	EP=25; WS=1; ITR=4	565	83.2	84.0	84.5	19.5
ADEs#3_SM	EP=25; WS=1; ITR=10	565	83.2	84.3	85.0	21.2
ADEs#3_SM	EP=5; WS=2; ITR=4	565	80.9	81.3	81.0	6.7
ADEs#3_SM	EP=5; WS=2; ITR=10	565	81.2	81.2	81.5	8.5
ADEs#3_SM	EP=25; WS=2; ITR=4	565	85.5	85.0	86.7	21.5
ADEs#3_SM	EP=25; WS=2; ITR=10	565	85.8	85.4	86.0	23.4
ADEs#3_Combined	EP=5; WS=1; ITR=4	21,843	87.5	88.6	88.0	61.7
ADEs#3_Combined	EP=5; WS=1; ITR=10	21,843	88.1	88.2	88.1	64.3
ADEs#3_Combined	EP=25; WS=1; ITR=4	21,843	90.0	89.4	89.5	116.5
ADEs#3_Combined	EP=25; WS=1; ITR=10	21,843	90.2	90.6	89.0	119.2
ADEs#3_Combined	EP=5; WS=2; ITR=4	21,843	88.3	88.2	88.5	62.5
ADEs#3_Combined	EP=5; WS=2; ITR=10	21,843	88.9	89.0	88.9	63.8
ADEs#3_Combined	EP=25; WS=2; ITR=4	21,843	92.1	92.0	93.2	118.2
ADEs#3_Combined	EP=25; WS=2; ITR=10	21,843	92.7	93.6	93.0	121.7

[a]EP: epoch.

[b]WS: window size.

[c]ITR: iteration.
